# Gambling Disorder in Minority Ethnic Groups

**DOI:** 10.1016/j.addbeh.2022.107475

**Published:** 2022-09-03

**Authors:** Jon E Grant, Samuel R Chamberlain

**Affiliations:** 1 University of Chicago, USA; 2 University of Southampton, UK

## Abstract

**Background:**

Although the data on racial/ethnic associations with gambling disorder are limited, studies suggest that ethnicity may have associations with both symptom severity and psychosocial impairment linked to gambling disorder. Based on the current literature, we hypothesized that there would be a difference in gambling symptom severity, and co-occurring disorders, as a function of racial-ethnic group.

**Methods:**

475 adults (mean age = 47.6 (± 11.6) years; 54.3% females) with gambling disorder who had participated in clinical trials on pharmacotherapy or psychotherapy were included. Participants were assessed for gambling severity, comorbidities, health issues, quality of life and psychosocial functioning. Participants who self-identified as Black, Asian or Minority Ethnic (BAME) were compared to those who self-identified as white Caucasian (non-BAME).

**Results:**

The BAME group had significantly earlier age of gambling problems (and age at first gambling), and higher levels of disability. The two groups did not differ significantly in terms of current gambling disorder symptom severity, previous suicide attempt(s), quality of life, percent of salary in past year lost to gambling, or likelihood of having received treatment for gambling disorder in the past, nor in terms of having used Gamblers Anonymous.

**Conclusions:**

These data show that having gambling disorder and being from a minority racial-ethnic group was associated with significantly earlier age at first gambling, earlier age of gambling becoming problematic, and higher levels of disability, in clinical trial settings. Future work should further examine differences in the clinical features of gambling disorder in different minority groups in larger sample sizes, ideally also longitudinally, across a range of settings. Identification of the reasons/mechanisms for differences in age of onset and levels of disability may lead to new public health and treatment targets to minimize gambling harms.

## Introduction

Gambling is a psychiatric disorder characterized by persistent, recurrent maladaptive patterns of gambling behavior and functional impairment (American Psychiatric Association, APA, 2013). High rates of problematic gambling have been reported in racial-ethnic minority groups (Alegria et al., 2009; Okuda et al., 2016), and studies have also indicated that higher rates of gambling in certain racial-ethnic minority groups may be due to different cultural norms and attitudes towards gambling (Raylu et al., 2004; Kong et al., 2013).

While the majority of research regarding the phenomenology of gambling disorder has focused on white Caucasian samples, only a handful of studies have examined to what extent there are differences in the presentation of gambling disorder as a function of racial-ethnic groups (for review see Okuda et al., 2016). Of the studies examining racial/ethnic differences in gambling, one study reported higher rates of gambling disorder in black as opposed to white individuals, but racial and ethnic groups generally had similar symptom patterns, time courses, and rates of treatment seeking (Alegria et al., 2009). Another study found that African-American gamblers were more likely to report co-occurring hypomania, a substance use disorder, or a mood disorder compared to white Caucasian gamblers (Barry et al., 2011). In a prior small study in non-treatment seeking young adults with gambling disorder (n=62), black individuals reported more symptoms of disordered gambling and higher self-report obsessive-compulsive traits than white individuals (Chamberlain et al., 2016). In a study of people calling into a gambling helpline, African-American individuals were more likely than Caucasian individuals to report longer duration of gambling problems, but were less likely to report having received mental health treatment (Barry et al., 2008). Finally, a large study of university students (n = 3058) found that Asian participants gambled less frequently than Caucasians or Hispanic/Latino(a)s, but spent more money overall on gambling than participants who were Black or Hispanic/Latino(a) (Rinker et al., 2016).

While examining how gambling (and gambling disorder) may differ across specific racial-ethnic groups is important, there is also a strong rationale for considering associations with minority racial-ethnic status *per se*; i.e. in Black, Asian, and Minority Ethnic (BAME) status. BAME status has often been linked in the literature to health inequalities, discrimination, and barriers to accessing support and treatment for mental health conditions (McGuire & Mirnda, 2008; Bhui et al., 2018; Hackett et al., 2020). In a review of the available data, concerningly high rates of gambling disorder were reported in BAME groups, but it was also noted that there were methodological issues in the literature and that studies were few in number (Okuda et al., 2016).

Therefore, the goal of this study was to better understand how minority ethnic (BAME) status may relate to clinical presentation, symptom severity and psychosocial impairment in a sample of adults with gambling disorder. Based on the current literature, we hypothesized that BAME individuals, compared to non-BAME individuals, would experience more severe gambling symptoms, and higher levels of co-occurring disorders.

## Methods

### Participants

475 adults with gambling disorder who had participated in clinical trials on pharmacotherapy or psychotherapy were included. The mean age was 47.6 (± 11.6) years and 54.3% were females. The current study used a combined database from ten published studies [Kim et al. (2001, 2002), Grant and Potenza (2006), Grant et al. (2003, 2006, 2007, 2009, 2010a, 2010b, 2013)] as well as on-going studies. Any participant who was in more than one clinical trial was only included once for purposes of these data and that was their initial visit.

Inclusion criteria for all studies were largely similar: current gambling disorder according to the Diagnostic and Statistical Manual of Mental Disorder 5 – DSM 5 (American Psychiatric Association, 2013) (subjects recruited before 2013 met DSM-IV criteria for pathological gambling and were retrospectively examined using the DSM-5 criteria) and the ability to understand the study and the consent form. The main change from DSM-IV to DSM-5 was removal of the ‘illicit act(s)’ criterion and so we were able to simply check if people meeting DSM-IV criteria also met DSM-5 criteria, in which case they were included. Exclusion criteria were: bipolar I disorder, schizophrenia, or substance use disorder within the last 3 months. Data at baseline (first visit) were used for the current study. The sample was enlisted in the metropolitan areas of Chicago, IL and Minneapolis, MN through advertisements on the internet, public places and newspapers. Participants were compensated with a gift card to local department stores.

After receiving a complete description of the study, participants provided written informed consent. All procedures involving human subjects were approved by the Institutional Review Boards at the University of Chicago and University of Minnesota.

### Measures

Participants were assessed for age, gender, marital status, educational level and racial-ethnic group (participants self-identified their racial group based on a single open-ended question). In addition, a semi-structured clinical interview was used to examine the clinical features of gambling disorder.

Clinical interviews were undertaken by trained raters using the Structured Clinical Interview for DSM (First et al., 2015), the Structured Clinical Interview for Gambling Disorder (SCI-GD) (Grant et al., 2004), and the Minnesota Impulse Disorders Inventory (MIDI) (Grant, 2008; Chamberlain and Grant, 2018). The MIDI is a structured clinical interview designed to assess whether diagnostic criteria are met for impulsive disorders such as trichotillomania, kleptomania, and binge-eating disorder.

Gambling severity was assessed using the Gambling Symptom Assessment Scale (GSAS) a 12-item self-report questionnaire that was developed at the time of our initial clinical trial in 2001 (Kim et al., 2009). The items assess urges; gambling involvement (time, frequency, duration, control); anticipatory excitement/tension; pleasure in gambling; emotional and personal problems due to gambling behavior. The final score ranges between 0 and 48 (Kim et al., 2009). Quality of life was examined using the Quality of life Inventory (QOLI) a 17-item self-administered scale that examines the person's quality of life in different areas (Frisch et al., 1992), and overall psychosocial functioning was quantified using the Sheehan Disability Scale (SDS) (Sheehan, 1983).

### Data Analysis

Prior to analysis, participants were classified into two groups: those who self-identified as Black, Asian or Minority Ethnic (BAME) and those who self-identified as white Caucasian (non-BAME). Demographic and clinical characteristics between the groups were compared using independent sample t-tests for continuous variables and Pearson’s chi-square tests for categorical variables (or Fisher’s exact test for cell sizes <5, per convention). Statistical significance was defined as p<0.05 uncorrected. We also reported effect sizes for significant results and the 95% confidence intervals.

The aim of the power calculation was to show that the intended sample size was appropriate to detect an effect size of minimal interest; and also consider whether statistical correction would be inappropriate at that sample size. This was based on an a priori power calculation: our total target sample size was around 500 participants and we expected the number of BAME participants to be approximately 10% i.e. 50 people. The expected sample size of n=50 in one group and n=450 in the other group was anticipated to yield 92% power to detect a difference of medium effect size (d=0.50) or larger based on this statistical threshold, two-tailed. All analyses were conducted using JMP Pro software. Statistical correction is not appropriate at this sample size. For example, even correcting to p<0.01 would yield less than 80% power to detect the effect size of minimum clinical interest (d=0.50).

## Results

The demographic and clinical measures from the two groups are presented in Table 1. In terms of demographic characteristics, it can be seen that the BAME and non-BAME groups did not differ in terms of age, education levels, or gender. In the BAME group, the distribution of racial-ethnic self-declared status was: African American (43.9% of group), Latino/Hispanic (19.51%), Asian (19.51%), Native American (12.20%), and other e.g. mixed race (4.88%).

Compared to the non-BAME group, the BAME group had significantly earlier age of gambling problems (Cohen’s D=0.55, 95% CI 0.18-0.91), earlier age at first gambling (D=0.47, 95% CI 0.08-0.85),, and higher levels of disability (D=0.30, 95% CI -0.02-0.30). The two groups did not differ significantly in terms of current gambling disorder symptom severity, prior suicide attempt(s), quality of life, percent of salary in past year lost to gambling, or likelihood of having received treatment for gambling disorder in the past, nor in terms of having used Gamblers Anonymous in the past.

There were no significant group differences in terms of overall percentage of people experiencing one or more mental health comorbidities (Table 1). Detailed breakdown of lifetime psychiatric comorbidity data are presented in Table 2.

There were no significant group differences in terms of overall percentages experiencing one or more physical health comorbidity, although there was a non-significant trend towards higher physical health morbidities in the BAME group (Table 1). Table 3 presents more detailed data on particular physical health comorbidities among adults with gambling disorder in the two groups.

## Discussion

There have been only a few studies focusing on associations of race/ethnicity with respect to gambling disorder (Okuda et al., 2016), and this study adds to the literature by demonstrating that several differences between the two study groups were found. The striking differences seem to be that BAME individuals reported starting to gamble at a significantly younger age, have an earlier age when gambling first became problematic, and higher disability.

Our finding that minority groups were equally likely to have previously received formal treatment for gambling disorder (medication/therapy), or attended Gamblers Anonymous, is important. Interestingly, the percentage of BAME and non-BAME participants in the study who attended Gamblers Anonymous was fairly low (approximately 30%) and those receiving formal treatment (such as medication or psychotherapy, whether outpatient or inpatient) was very low (approximately 10-15%). In keeping with our results, the National Epidemiologic Survey on Alcohol and Related Conditions study found that rates of treatment seeking in gambling disorder did not differ significantly across minority and non-minority groups (Alegria et al., 2009). Contrary to the current study however, this prior study did not find significant differences in the course of gambling disorder (e.g. symptom onset). The prior study also did not identify significant differences in terms of current physical, mental, or social functioning. The reasons for these relative discrepancies across studies are unclear but could reflect different natures of the samples such as the ethnic distributions or methods of analyses. For example, the studies were addressing different statistical questions – we addressed whether BAME people differ to non-BAME overall, whereas the previous study addressed whether specific ethnic groups differed from each other.

There are several limitations to the current study. The study was not pre-registered. We focused our analysis on comparing BAME to non-BAME individuals. We acknowledge that this terminology/definition itself is not universally accepted; however, we suggest it may be a useful starting point to better understand how certain minority groups may differ in the clinical features/presentation of gambling disorder. We did not report or examine variables in different minority groups as the sample sizes would have been too small to be meaningful, and the study was neither designed nor powered to do so. Also, we did not collect all possible demographic measures. Future work may wish to examine whether the clinical presentation of gambling disorder differs in specific racial/ethnic groupings, rather than the broader category of BAME, and effects of other demographic measures. Because the dataset was pooled from previous and on-going clinical trial studies (treatment trials), the data may not generalise to individuals with gambling disorder who do not participate in treatment trials and/or are not treatment seeking. Another limitation is that the data are cross-sectional, and so causality cannot be ascribed. Also, we included people meeting DSM-IV or DSM-5 criteria. While we only included participants meeting DSM-5 symptoms (i.e. we accounted for removal of the ‘illegal acts’ criterion) it was not possible to ensure all those included would have met DSM-5 full requirements in terms of the exact phraseology, since the DSM-5 was not available at the time). Power would have been limited to detect more subtle differences between BAME and non-BAME groups, particularly for categorical data occurring with low frequencies. Finally, since the pooled dataset included clinical trials in which comorbid substance use disorder(s) was/were exclusionary, it is important to consider that findings may differ in people with gambling disorder who do have these conditions or are not taking part in clinical trials. There is a possibility of differential exclusion if minority status was linked to rates of substance use disorders and it should be noted that minority groups are often under-represented (relative to census data) in clinical trials *per se* (Rochon et al., 2004). Many, but not all studies, suggest lower rates of substance use disorders in most minority ethnic groups (i.e. relative to non-minority groups) (see e.g. Jackson et al., 2004; Wu et al., 2011; Hasin and Grant, 2015). As such effects of this exclusion criterion (i.e. substance use disorders) as a function of racial status remain speculative. However, theoretically, if substance use disorders are generally lower in minority groups viewed collectively, this could have led to disproportionate exclusion of non-BAME individuals in the current study.

In conclusion, this study found that in people with gambling disorder taking part in treatment trials, being from a minority racial-ethnic group was associated with significantly earlier age at first gambling, and earlier age of gambling becoming problematic, as well as with higher levels of disability. Future work should further examine differences in the clinical features of gambling disorder in different minority groups in larger sample sizes, including specific racial-ethnic categories, ideally also longitudinally. It would also be of value to explore the influence of whether people are first or second generation immigrants or not, bearing in mind findings in other areas of mental health (e.g. Coid et al., 2008). The novel finding of earlier onset of gambling and high disability in BAME people with gambling disorder may have implications for minimizing public health harms from gambling as well as developing more tailored interventions in future. For example, research could explore the reasons for earlier onset of gambling disorder in minority groups – for example, is it due to different exposure to gambling cues or opportunities, for example? If so that may suggest changes to legislation or treatments that might help minimize gambling harms.

This paper also highlights the importance of ensuring people from minority groups have access to mental health support and care for gambling disorder, given the elevated rate of disability observed in BAME individuals.

## Figures and Tables

**Table 1:** Demographic and clinical characteristics of the two groups.

	Non-BAME (N=434)		BAME (N=41)		chi-sq / t-test	p
	N	Column %	N	Column %		
	*Mean*	*SD*	*Mean*	*SD*		
**Age**	*47.77*	*11.57*	*45.61*	*12.24*	*-1.085*	*0.256*
**Education Level**	*2.89*	*1.04*	*2.73*	*1.07*	*-0.917*	*0.364*
**Sex**					1.150	0.284
Female	239	55.07%	19	46.34%		
Male	195	44.93%	22	53.66%		
**Age when started gambling, year**	*30.03*	*14.09*	*22.39*	*12.64*	*-3.204*	*0.003*
**Age when gambling became a problem, years**	*38.87*	*12.81*	*32.93*	*12.19*	*-2.474*	*0.019*
**Percent income lost to gambling, past year**	*87.58*	*284.55*	*61.97*	*84.41*	*-0.972*	*0.335*
**Gambling Symptom Assessment Scale, total score**	*35.91*	*12.75*	*34.90*	*10.16*	*-0.506*	*0.616*
**Previously went to Gamblers Anonymous?**					1.116	0.291
No	282	64.98%	30	73.17%		
Yes	152	35.02%	11	26.83%		
**Previously had formal treatment for Gambling Disorder?**					#	*0.3705*
**No**	365	84.1%	37	90.24%		
**Yes**	69	15.9%	4	9.76%		
**Quality of Life Inventory (T score)**	*32.94*	*15.07*	*33.00*	*11.92*	*0.013*	*0.990*
**Sheehan Disability Score (SDS)**	*15.22*	*6.65*	*17.22*	*7.12*	*2.351*	*0.0204*
**Number of previous psychiatric hospitalizations**					#	*0.373*
0	411	94.70%	39	95.12%		
1	20	4.61%	1	2.44%		
2	3	0.69%	1	2.44%		
**Any current psychiatric comorbidity?**					1.362	0.24
No	223	51.38%	25	61.00%		
Yes	211	48.62%	16	39.00%		
**History of suicide attempt(s) due to gambling?**					#	0.0743
No	177	98.88%	23	92.00%		
Yes	2	1.12%	2	8.00%		
**Any physical health morbidity?**					3.211	0.073
No	411	94.70%	36	87.80%		
Yes	23	5.30%	5	12.20%		

Note: some cell sizes are smaller if variable not available for all participants (cell sizes indicated).

# Indicates Fisher’s exact test.

**Table 2:** Lifetime Psychiatric Comorbidities in Gambling Disorder.

	Non-BAME		BAME	
	N	Column %	N	Column %
**Major depressive disorder**				
No	349	80.41%	33	80.49%
Yes	85	19.59%	8	19.51%
**Social Phobia**				
No	421	97.00%	41	100.00%
Yes	13	3.00%	0	0.00%
**Panic disorder**				
No	421	97.00%	40	97.56%
Yes	13	3.00%	1	2.44%
**Adjustment disorder**				
No	430	99.08%	41	100.00%
Yes	4	0.92%	0	0.00%
**Any Anxiety Disorder**				
No	394	90.78%	39	95.12%
Yes	39	8.99%	2	4.88%
**Bulimia nervosa**				
No	432	99.54%	41	100.00%
Yes	2	0.46%	0	0.00%
**Binge-eating disorder**				
No	422	97.24%	41	100.00%
Yes	12	2.76%	0	0.00%
**Trichotillomania**				
No	425	99.30%	41	100.00%
Yes	2	0.47%	0	0.00%
**Compulsive sexual behaviour disorder**				
No	417	97.43%	40	97.56%
Yes	11	2.57%	1	2.44%
**Kleptomania**				
No	422	98.60%	40	97.56%
Yes	6	1.40%	1	2.44%
**Intermittent explosive disorder**				
No	427	99.77%	41	100.00%
Yes	1	0.23%	0	0.00%
**Compulsive buying disorder**				
No	408	95.10%	38	92.68%
Yes	21	4.90%	3	7.32%

**Table 3:** Physical health comorbidities in the two groups.

	Non-BAME		BAME	
	N	Column %	N	Column %
**None**	411	94.70%	36	87.80%
**Hypertension**	12	2.76%	3	7.32%
**Diabetes**	1	0.23%	0	0.00%
**Hypertension and diabetes**	1	0.23%	0	0.00%
**Hypertension and diabetes and obesity**	1	0.23%	0	0.00%
**Diabetes**	5	1.15%	1	2.44%
**Gastric bypass**	2	0.46%	0	0.00%
**Asthma**	1	0.23%	1	2.44%
